# Conserved core microbiota in managed and free-ranging *Loxodonta africana* elephants

**DOI:** 10.3389/fmicb.2023.1247719

**Published:** 2023-10-04

**Authors:** Milan Thorel, Dasiel Obregon, Baptiste Mulot, Apolline Maitre, Lourdes Mateos-Hernandez, Pierre-Yves Moalic, Alejandra Wu-Chuang, Alejandro Cabezas-Cruz, Antoine Leclerc

**Affiliations:** ^1^ZooParc de Beauval and Beauval Nature, Saint-Aignan, France; ^2^School of Environmental Sciences, University of Guelph, Guelph, ON, Canada; ^3^Anses, INRAE, Ecole Nationale Vétérinaire d’Alfort, UMR BIPAR, Laboratoire de Santé Animale, Maisons-Alfort, France; ^4^Labofarm Finalab Veterinary Laboratory Group, Loudéac, France

**Keywords:** elephant, zoo, *Loxodonta africana*, microbiome composition, microbiota, Proboscidae

## Abstract

The gut microbiota plays a crucial role in animal health and homeostasis, particularly in endangered species conservation. This study investigated the fecal microbiota composition of European captive-bred African savanna elephants (*Loxodonta africana*) housed in French zoos, and compared it with wild African savanna elephants. Fecal samples were collected and processed for DNA extraction and amplicon sequencing of the 16S rRNA gene. The analysis of α and β diversity revealed significant effects of factors such as diet, daily activity, and institution on microbiota composition. Specifically, provision of branches as part of the diet positively impacted microbiota diversity. Comparative analyses demonstrated distinct differences between captive and wild elephant microbiomes, characterized by lower bacterial diversity and altered co-occurrence patterns in the captive population. Notably, specific taxa were differentially abundant in captive and wild elephants, suggesting the influence of the environment on microbiota composition. Furthermore, the study identified a core association network shared by both captive and wild elephants, emphasizing the importance of certain taxa in maintaining microbial interactions. These findings underscore the impact of environment and husbandry factors on elephant gut microbiota, highlighting the benefits of dietary enrichment strategies in zoos to promote microbiome diversity and health. The study contributes to the broader understanding of host-microbiota interactions and provides insights applicable to conservation medicine and captive animal management.

## 1. Introduction

The intricate relationship between the gut microbiota (GM), which contains trillions of microorganisms (Budd et al., 2020), and the health of organisms is an emerging frontier in biology, and its significance extends even to the realm of conservation medicine for endangered species (Bahrndorff et al., 2016; West et al., 2019). Conservation efforts often focus on external factors such as habitat protection, breeding programs, and disease management. However, the internal microbial communities residing within animals, particularly their digestive tracts, are increasingly recognized as pivotal players in the overall health and well-being of these species (Bahrndorff et al., 2016; West et al., 2019). Endangered species often face challenges that disrupt their natural environments, alter their diets, and expose them to new pathogens. These disruptions can impact the balance of the GM, leading to health issues and reduced reproductive success (Lee and Hase, 2014; Shreiner et al., 2015; Keady et al., 2021). The GM plays a primary role in many physiological processes, including digestion and nutrient uptake, immunity, or reproduction, and dysbiosis (i.e., deviations from balanced microbiota) can lead to many pathological processes (Kau et al., 2011; Lee and Hase, 2014; Shreiner et al., 2015; Fabbiano et al., 2017; Keady et al., 2021). Accordingly, by studying the GM of endangered species, researchers can gain insights into how these microorganisms contribute to the overall health and adaptability of the animals. This understanding can inform conservation efforts by guiding dietary recommendations, disease management strategies, and even assisted reproductive techniques (Keady et al., 2021).

Elephants are non-ruminant, monogastric herbivorous, hindgut fermenters (Van Hoven and Lankhorst, 1981; Dumonceaux, 2006). In the wild, African savanna elephants (*Loxodonta africana*) have preferences in plant types and parts, and diet selection is optimized for energy, growth or reproduction (Biru and Bekele, 2012; Pretorius et al., 2012; Mwambola et al., 2014; Sach et al., 2019). As such, zoo-housed elephants should be provided with various types of hay and browse with differing energetic and digestibility coefficients (Bolechova et al., 2020). As diet has a direct influence on the gut microbiota of African herbivores (Kartzinel et al., 2019), GM differences between managed vs. free-ranging elephants are expected. Elephant fermentation primarily occurs in the colon, even if some fermentative bacteria can be found in their cecum (Van Hoven and Lankhorst, 1981; Clauss et al., 2003; Dumonceaux, 2006). As a consequence, the highest apparent digestibility is reported in the upper portion of their colon (Clemens and Maloiy, 1983), and energy supply is based on short-chain fatty acids originating from colonic fermentation of fibrous forage (Greene et al., 2019a).

Elephant dung boluses are easy to collect, non-invasive samples whose composition is a good predictor of their hindgut health, and the fecal/hindgut microbiome is often used as a proxy of the GM microbiome (Budd et al., 2020; Kandel et al., 2020; Keady et al., 2021; Moustafa et al., 2021). As other colonic fermenters with similar digestive physiology, equids are often used as a model for digestion in Proboscidae, and their GM has already been described (Kauter et al., 2019). Nevertheless, some differences exist (Clauss et al., 2003; Greene et al., 2019a). In particular, elephants exhibit faster ingesta passage rates, leading to lower nutrient digestibility coefficients (Hackenberger, 1987; Clauss et al., 2003). To optimize energy intake, elephants are able to increase food intake without dramatically raising ingesta passage (Clauss et al., 2007). These differences with horses reinforce the need for a precise description of the GM in elephants. Some data is available for wild African elephants (Budd et al., 2020), and one recent study evaluated the influence of clinical and hormonal findings in African savanna elephants housed in US institutions (Keady et al., 2021), but to the best of the authors’ knowledge the influence of husbandry and individual factors on the fecal microbiota of zoo-managed African savanna elephants is not available, nor is a comparison between captive and wild elephants.

The pursuit of the core microbiome has gained prominence in microbial ecology, shedding light on the interactions between microbial communities and their hosts or environments (Neu et al., 2021). Essentially, a core microbiome encompasses shared microbial taxa and their associated genomic or functional attributes, characterizing specific hosts or ecosystems (Turnbaugh et al., 2007; Hamady and Knight, 2009; Risely, 2020). This endeavor has prompted investigations into genes (Turnbaugh et al., 2009), functional pathways (Jiang et al., 2016), and metabolic profiles (Stefanini et al., 2017) common among microbial communities in various settings. Typically, uncovering a core microbiome involves identifying shared taxa across multiple microbial communities within a given host species or environment, hypothesizing these shared taxa represent vital microbial associates of the host or environment under the conditions studied (Shade and Handelsman, 2012). These core members are assumed to have ecological and functional importance, with potential implications ranging from human health (Zaura et al., 2009; Bäckhed et al., 2012) to responses to climate change (Ainsworth and Gates, 2016; Hutchins et al., 2019) leading to a surge in core microbiome studies (Neu et al., 2021). However, core microbiome analyses differ in their criteria to define the core. The process typically involves determining the proportion of samples sharing a set of microbial taxa, their relative abundances, or a combination of both. This quantification spans different taxonomic levels, from amplicon sequence variants (ASVs) to phyla, and across various spatial and temporal scales (Neu et al., 2021). More recently, core association networks (CAN) were proposed (Röttjers et al., 2021) as a novel approach to examine the concept of the core microbiome. These networks extend the understanding of microbial interactions beyond individual taxa by investigating the associations and co-occurrences among them. A core association network represents a subset of microbial interactions that are conserved across different samples or environments, providing insights into the shared microbial partnerships that might underlie core microbial communities (Röttjers et al., 2021).

The objectives of the present study were (1) to characterize the fecal microbiota of European captive-bred African savanna elephants based on amplicon sequencing (16S rRNA) of dung samples, (2) to measure the influence of several factors (age, sex, daily activity, residence zoo, type of diet including provision of branches, birthplace, and social status), and (3) to compare its composition and diversity to previously reported data in free-ranging African savanna elephants. We hypothesized that (a) captive and wild animals of the same species develop different microbiomes due to their differing environments, (b) individual and environmental factors affect microbiome composition, and proper diet and exercise correspond with a healthier microbiome. If these hypotheses hold true, we should observe that (a) the microbiota of captive-bred African savanna elephants differs from that of their wild counterparts, (b) differences are observed as a function of influence factors, and (c) branch intake and diversity and increased exercise lead to positive health adaptations.

## 2. Materials and methods

### 2.1. Animals and study design

All African savanna elephants living in French zoological institutions and listed in the Zoological Information Management System (ZIMS, Species360, Bloomington, MN 55425, United States) were included (*n* = 21), representing seven institutions. All elephants (8 males, 13 females) had access to indoor and outdoor enclosures. Two elephants were solitary and the remaining 19 were living in groups of two or more. Table 1 shows the breakdown of the subjects by zoo. Husbandry factors were specific to each institution (diet, zootechny, enclosure size, reproduction strategies, training). Gathered data included institution (zoological park of residence), sex, age, birthplace (in captivity or in the wild), daily activity (approx. Kilometers per day), diet (including provision of branches or not), and social status (in group or solitary). An age class was attributed to each elephant (juvenile: <5 yrs.; adolescent: [5–10] yrs.; sub-adult: [10–20] yrs.; adult: [20–30] yrs.; mature adult: [30–40] yrs.; old: [40–50] yrs.; geriatric: [50–60] yrs). Daily activity was classified as low (<5 km daily) vs. high (>5 km). Diets were sorted in six groups depending on specific food items provided to elephants secondary to hay (grass and alfalfa): V-S (vegetables + seeds), C-B (carrots + bread), H-C (pellets for horses and cattle), E-C (pellets for elephants and cattle), F-S (fruits and seeds), F-V (fruits and vegetables). Vegetables included greens, fruits included apples and bananas, seeds included sunflower seeds or peanuts mainly, pellets were branded-formula specific to horses, cattle, or elephants. The provision of branches (including hornbeam, bamboo, willow, birch, oak and/or chestnuts) was also recorded separately.

**Table 1:** Zoo African savanna elephants study population.

Zoo ID	Elephant ID	Born		Sex		Age		Provision of browse		Daily activity			Social	
		Captive	Wild	*F*	*M*	<20 yr	>20 yr	+	−	<5 km	>5 km	Unknown	In group	Solitary
7	2,145	1	0	0	1	1	0	0	1	1	0	0	1	0
7	2,144	1	0	1	0	0	1	0	1	1	0	0	1	0
7	2,143	0	1	1	0	0	1	0	1	1	0	0	1	0
7	2,142	0	1	1	0	0	1	0	1	1	0	0	1	0
Total zoo 7		**2**	**2**	**3**	**1**	**1**	**3**	**0**	**4**	**4**	**0**	**0**	**4**	**0**
6	2,141	0	1	1	0	0	1	1	0	0	1	0	1	0
6	2,140	1	0	1	0	1	0	1	0	0	1	0	1	0
6	2,139	0	1	1	0	0	1	1	0	0	1	0	1	0
6	2,138	0	1	1	0	0	1	1	0	0	1	0	1	0
6	2,137	0	1	1	0	0	1	1	0	0	1	0	1	0
6	2,136	0	1	1	0	0	1	1	0	0	1	0	1	0
Total zoo 6		**1**	**5**	**6**	**0**	**1**	**5**	**6**	**0**	**0**	**6**	**0**	**6**	**0**
5	2,135	1	0	0	1	1	0	1	0	0	1	0	1	0
5	2,134	1	0	0	1	1	0	1	0	0	1	0	1	0
Total zoo 5		**2**	**0**	**0**	**2**	**2**	**0**	**2**	**0**	**0**	**2**	**0**	**2**	**0**
4	2,133	0	1	0	1	0	1	1	0	1	0	0	0	1
Total zoo 4		**0**	**1**	**0**	**1**	**0**	**1**	**1**	**0**	**1**	**0**	**0**	**0**	**1**
3	2,132	1	0	0	1	0	1	1	0	0	1	0	1	0
3	2,131	0	1	1	0	0	1	1	0	1	0	0	1	0
Total zoo 3		**1**	**1**	**1**	**1**	**0**	**2**	**2**	**0**	**1**	**1**	**0**	**2**	**0**
2	2,130	1	0	0	1	1	0	0	1	1	0	0	1	0
2	2,129	1	0	0	1	1	0	0	1	1	0	0	1	0
Total zoo 2		2	0	0	2	2	0	0	2	2	0	0	2	0
1	2,128	1	0	0	1	1	0	1	0	0	0	1	0	1
1	2,127	1	0	1	0	0	1	1	0	0	0	1	1	0
1	2,126	0	1	1	0	0	1	1	0	0	0	1	1	0
1	2,125	1	0	1	0	1	0	1	0	0	0	1	1	0
Total zoo 1		**3**	**1**	**3**	**1**	**2**	**2**	**4**	**0**	**0**	**0**	**4**	**3**	**1**
Total		**11**	**10**	**13**	**8**	**8**	**13**	**15**	**6**	**8**	**9**	**4**	**19**	**2**

Figures in bold indicate the total number of elephants in each category for each zoo.

### 2.2. Fecal samples collection and optimization of sample conservation protocol

A preliminary study was conducted to select the most reliable and convenient sampling and storage method for the main study (Supplementary Figure 1). Six fecal samples from four captive-bred female African savanna elephants were collected in a sterile manner. Each of the six samples was stored using four methods: (1) fresh in a sterile tube (+4°C), (2) in a sterile tube containing RNAlater (+20°C), (3) on FTA cards (filter papers containing chemicals that denature proteins, lyse cells and protect nucleic acids, +20°C), and (4) by desiccation on silica gel (+20°C). Briefly, the desiccation protocol consisted of placing 10.1 g of sterile feces in 70% ethanol. After 24 h, the ethanol was removed and the feces were recovered in a sterile gauze, that was then placed in a sterile tube containing silica beads. Samples were sent at +4°C to the laboratory 48 h after sampling, and DNA extraction and sequencing were performed as described below. More bacterial taxa were sequenced from fecal samples stored on FTA cards or in RNAlater compared to fresh feces and the dessication method (phylum level, Supplementary Figure 1a). Using PCoA analyses a better bacterial differentiation was seen for the method using FTA-cards (ellipse of larger surface) compared to the three other methods (fresh feces, RNAlater, dessication; Supplementary Figure 1b). Shannon indexes were significantly different between sampling methods (*p* = 0.019), and the highest Shannon and Simpson indexes were seen for FTA cards (Supplementary Figure 1c). FTA cards were therefore chosen as the sampling method for the main study.

For each elephant, a fresh, integer fresh dung bolus was identified and gently opened with clean and disinfected (chlorine bleach) gloves without contaminating its inner part. The center of each bolus of dung was sterile sampled as previously described (Zhang et al., 2023). This time, a sterile swab was placed in its center and firmly pressed onto the fecal material. The swab provided an easy-to-take, sterile, uncontaminated sample, and the pressure exerted by the handler on the swab inside the dung bolus allowed its liquid fraction to soak it, as the moisture content of elephant dungs was previously estimated as more than 49% (Stępień et al., 2019). Once totally wet, the swab was dropped in the center of an FTA-card and gently rolled over within the spot, to well impregnate the blotting paper. All samples were taken between June 2020 and early October 2020. The FTA cards were stored at room temperature (+20°C maximum) and sent to the laboratory for DNA extraction and sequencing.

### 2.3. DNA extraction

Genomic DNA was extracted from FTA cards using a MoBIO PowerSoil DNA isolation kit (Qiagen GmbH, Hilden, Germany) following manufacturer’s instructions. Briefly, the spots were cut and left soaked in physiological water during 15 min under gentle agitation. A volume of 200 μL of the suspension was then used for DNA extraction. DNA quantity and quality were examined using Qubit 2.0 Fluorometer™ (Thermo Fisher Scientific, Waltham, United States) according to the manufacturer’s instruction. DNA extracts were then kept at −20°C until analysis.

### 2.4. Library preparation and sequencing

The Ion 16S Metagenomics Kit (Thermo Fisher Scientific, Waltham, United States) was used to amplify the V2, V3, V4, V6-7, V8, and V9 hypervariable regions of 16S rRNA gene from prokaryotes using two amplification reactions, based on two primer pools targeting the region V2–4–8 and V3–6, 7–9, respectively. The Ion Plus Fragment Library Kit (Thermo Fisher Scientific, Waltham, United States) was used to ligate barcoded adapters to the generated amplicons and create the barcoded libraries. Template preparation of the created amplicon libraries was done on the automated Ion Chef System using the Ion 520TM/530TM Kit-Chef (Thermo Fisher Scientific, Waltham, United States) according to the manufacturer’s instructions. Sequencing was carried out on an Ion 530 chip using the Ion S5 platform (Thermo Fisher Scientific, Waltham, United States). Data (bam file) were exported to Ion Reporter software 5.2 (Workflow version: 1.1; Thermo Fisher Scientific).

### 2.5. External data set from wild African savanna elephants

The 16 s rRNA sequence data from free-ranging African elephants was already decribed by a previous study (Budd et al., 2020), and is available online at NCBI-SRA (Sequence Read Archive, accession: PRJNA587772). Briefly, the data set included 48 female and male African elephants, 35 savanna elephants (*Loxodonta africana*) from the Transmara and Narok districts in southwestern Kenya and 13 forest elephants (*Loxodonta cyclotis*) from Lope National Park in Gabon. However in this study we only used the samples from the savanna animals, that comprised 18 and 17 female and male elephants respectively, from the age groups juvenile (*n* = 4; two females and two males), subadult (*n* = 13; seven males and six males) and adult (*n* = 18; nine individuals from each sex). This data set was produced using the Illumina MiSeq technology, targeting the variable region V4, using the universal primers 515F (5′-GTG CCA GCM GCC GCG GTA-A3’) /806R (5′-GGA CTA CHV GGG TWT CTA AT-3′).

### 2.6. 16S rRNA sequences data processing

All sequence processing in this study was conducted using the QIIME 2 pipelines (Bolyen et al., 2019). The sequences were denoised using the DADA2 approach, which resolves each amplicon sequence variant (ASV) (Callahan et al., 2016), and the taxonomy assignment was based on the 16S rRNA SILVA database v.138 (Quast et al., 2013). However, different bioinformatic pipelines (steps) were used for each dataset, as the two sets were produced by different sequencing technologies. Specifically, 16S amplicon sequencing from zoo elephants was produced with Ion Torrent technology, while the wild elephants samples were sequenced using Illumina technology. This required distinct processing approaches to accommodate the unique characteristics of each technology.

The zoo sequencing data (zoo data), which included short-read amplicon sequences from multiple regions (V2, V3, V4, V6-7, V8, and V9), were processed individually and then combined in the reconstruction of a near full-length 16S marker gene using the Short Multiple Reads Framework (SMURF) algorithm (Fuks et al., 2018), wich was used as implemented QIIME2 plugin q2-sidle (Debelius et al., 2021). Briefly, the raw sequences were separated into sets for each V region, using the q2-cutadapt plugin. Only forward sequences were used due to the verified overlap of forward and reverse sequences for each targeted amplicon region. Each V region (sequences package) was denoised using q2-dada2 plugin. Then, the Silva database, previously curated using RESCRIPt (Robeson et al., 2021), was used. The exact sequence fragments (by regions) were extracted from SIlVA sequences using qiime2 feature-classifier (extract-reads) plugin. Using q2-sidle, each set of ASVs and the respective SIlVA fragments sequences were aligned forming kmers, then the kmer were mapped with full-length SILVA reference sequences to reconstruct the taxonomic profile.

The wild sequencing data (wild data) was dowloaded for the SRA using q2-fondue pluging in QIIME2. Demultiplexed paired-end sequences were analyzed using via q2-dada2 for denoising and merging paired reads. The ASVs were aligned using the MAFFT (Katoh et al., 2002) via q2-alignment plugin, and the alignments were then used to construct the phylogeny following the FastTree2 method (Price et al., 2010) as implemented in q2-phylogeny. The taxonomic classification of the ASVs was performed using the Classify-Sklearn Naive Bayes method via the q2-feature-classifier plugin, based on SILVA database v.138 (Quast et al., 2013).

### 2.7. Statistical analyses

Comparisons of fecal microbiota were made (i) between groups of zoo elephants (according to influence factors) and (ii) between the entire group of zoo elephants and the wild elephants, for which the taxonomic tables from both data sets were combined, and collapsed at genera level. α diversity metrics (observed features, Pielou evenness index, and Shannon entropy) and β diversity (Bray Curtis’s distance) were compared, based on rarefied tables, using the Kruskal–Wallis test and the Permanova test, respectively.

In addition, we determined the “core microbiota” among zoo and wild elephants, which refers to any set of microbial taxa, or the functional attributes associated with those taxa, that are characteristic of a host or environment of interest (Neu et al., 2021). We aimed to identify those taxa ubiquitous in almost all the samples from both datasets, and for this we used the method described by Chong et al. (2020). This approach identifies the specific taxa by considering their prevalence across the entire set of data, which includes all samples from wild and zoo elephants. Furthermore, this method also determines when the shared taxa are abundant across the dataset by analyzing their relative abundance. In our study, we considered core taxa those genera that were present in at least 70% of the samples. These analyses were performed using the web tool MicrobiomeAnalyst, accessible at: https://www.microbiomeanalyst.ca (Chong et al., 2020).

The differences in taxonomic composition between groups were tested using a Kruskal–Wallis test as implemented in ALDEx2 method (Fernandes et al., 2013) on R studio (R Foundation for Statistical Computing). The ALDEx2 method entails creating Monte Carlo samples of Dirichlet distributions to account for the uncertainty in the number of reads in each sample, then transforming the data using the centered log ratio (clr) transformation (Aitchison, 1986), allowing standard statistical methods to be used. The detected differentially abundant taxa were used to create a heatmap in R studio (R Foundation for Statistical Computing).

#### 2.7.1. Bacterial co-occurrence networks and core association network

Co-occurrence networks provide a visual representation of the relationships among bacterial genera in a dataset. In these networks, the “nodes” represent the individual genera, and the “edges” symbolize the relationships between them, indicating either positive (weight > 0.50) or negative (weight < −0.50) correlations. The significance of these correlations in this study were determined using the Sparse Correlations for Compositional data (SparCC) method (Friedman and Alm, 2012) in R studio (R Foundation for Statistical Computing). The topological features of the networks further capture the structure and connectivity of the microbial community. These features include the number of nodes and edges, which describe the complexity of the network; “modularity,” referring to the degree to which the network can be divided into separate communities or clusters of interacting genera; “average degree,” indicating the average number of connections per node; and “weighted degree,” representing the strength of connections. Analyzing these aspects can provide insights into the ecological dynamics and stability of the microbial community. The visualization and analysis of these topological features were performed using Gephi v0.9.2 (Bastian et al., 2009).

The resulting networks were then analyzed to identify conserved patterns of microbial associations among both zoo and wild elephant populations. These conserved patterns are referred to as core association networks (CANs). CANs represent the stable and consistent relationships that are observed across different samples or conditions (Röttjers et al., 2021). To detect CANs, we employed the “Anuran” method, using its default parameters (full standalone). The Anuran method utilizes the null model technique, creating random networks in which links between taxa are assigned randomly. By comparing the actual data to these random models, Anuran is able to identify non-random patterns of associations that are conserved across various networks (Röttjers et al., 2021). The CAN analysis aimed to explore whether certain microbial associations persist consistently across captive and wild African savanna elephants. As these correlations are not expected to occur randomly, their presence suggests meaningful microbial associations, despite the differences in their living conditions.

#### 2.7.2. Modeling of networks robustness

To test the robustness of the networks, the connectivity loss of networks was calculated depending on removal of nodes by three methods [i.e., random removal, high degree removal first and cascading removal (high betweenness centrality, recalculated at each removal first)]. The “Network Strengths and Weaknesses Analysis” (“NetSwan”) script was used implemented in Rstudio (Lhomme, 2015).

## 3. Results

### 3.1. Diet predominantly influences the microbiota of African savanna elephants in zoos

**Figure 1:**
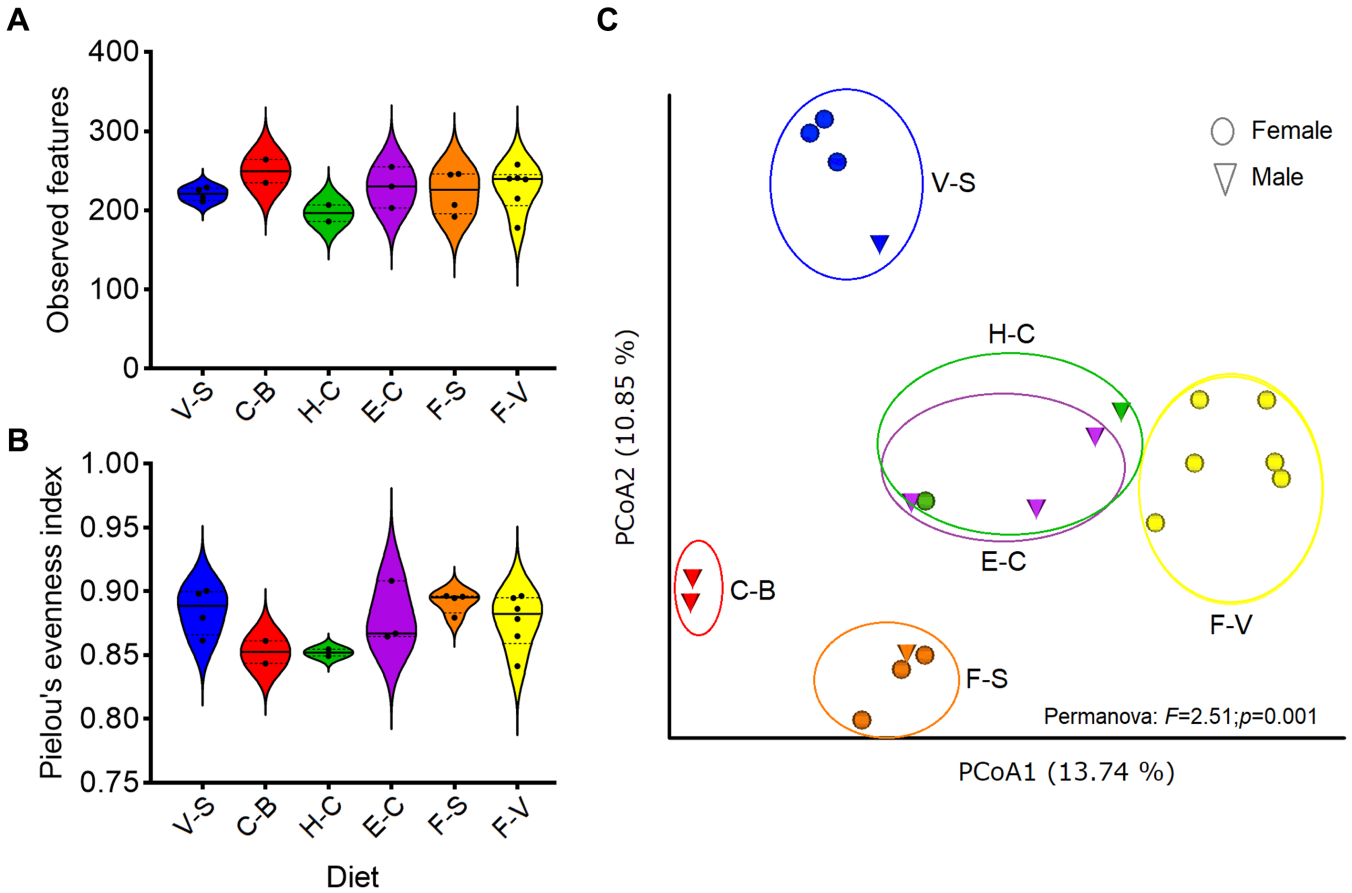
Diversity of zoo elephant microbiota with different diets. Between-group comparisons were performed on microbial richness **(A)** and evenness **(B)**, with no significant differences (Kruskal–Wallis, *p* > 0.05). **(C)** Bray Curtis distance was used to test for differences in microbiota composition among groups based on diets (Permanova, *p* < 0.05). The analyses were performed on rarefied feature tables at ASVs level.

No significant differences were detected in α diversity metrics (ASV level) when analyzing (Kruskal–Wallis test) each factor (diet, sex, age, etc) individually (Supplementary Table 1). No differences were found among diets (observed features: *H* = 5.2; *p* = 0.38, or Pielou index: *H* = 8.5; *p* = 0.12; Figures 1A,B). Higher α-diversity was seen with increased age (> 20 yr-old), but this was not significant (*p* = 0.85, Kruskall–Wallis test). However, differences were observed when comparing β diversity among groups of diets (Permanova, *p* = 0.001; Figure 1C). Pellet-based diets (H-C and E-C) were grouped together, regardless of the brand of pellets considered (designed for horses, cattle and/or elephants), showing the same relative abundance of bacterial taxa. Diets consisting solely of carrots and bread (“C-B,” excluding hay) showed the lowest bacterial abundance. A marked difference in bacterial taxon abundance was observed for fruits and vegetables, depending on whether or not they were associated with seeds (sunflower, peanuts).

**Figure 2:**
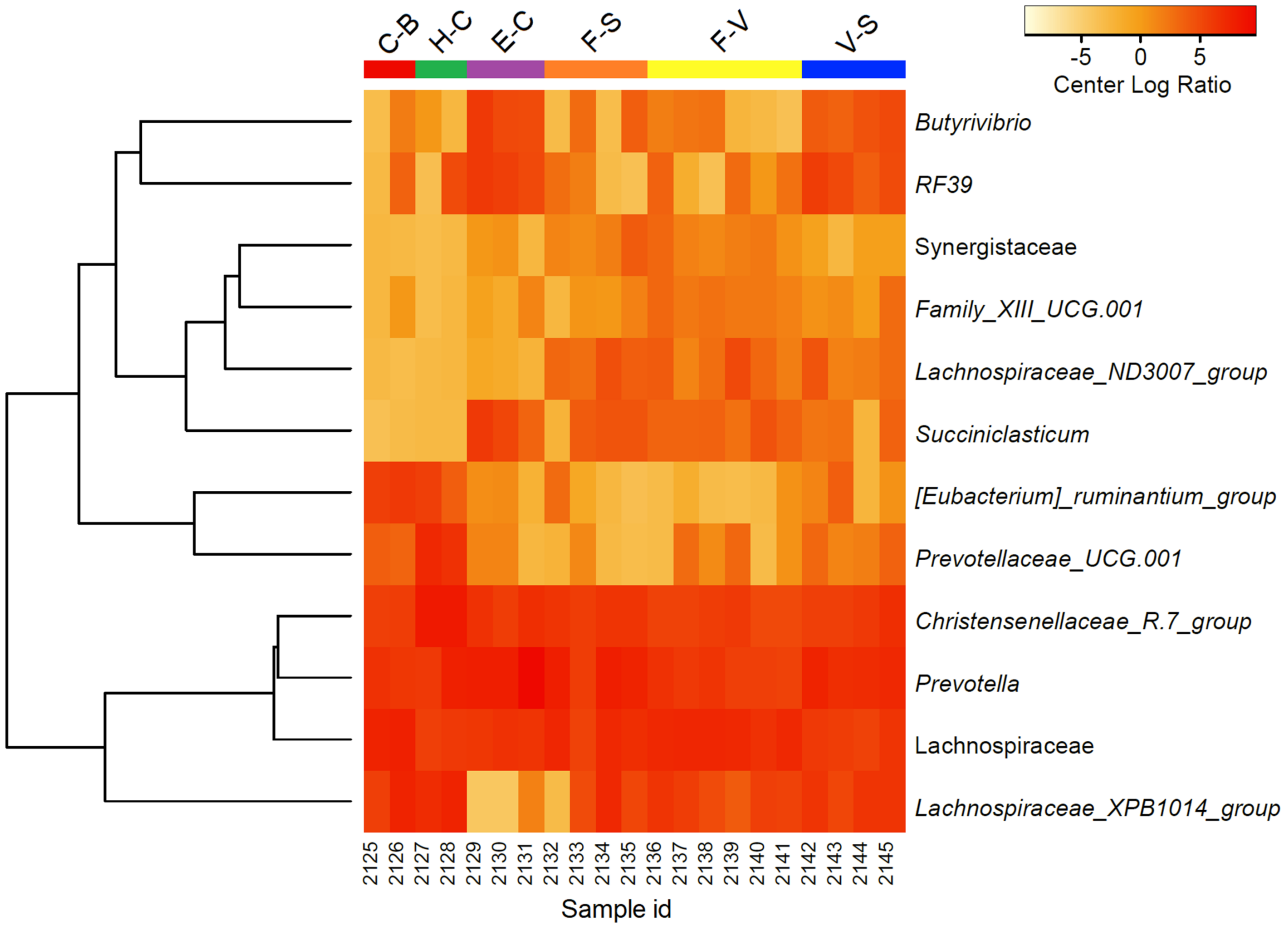
Differentially abundant microorganisms (genera level) detected among zoo elephants based on their diets. Only the 12 top significantly different taxa were included in the heatmap (Aldex, Welch test: *p*_adj_ < 0.05). Abundance profiles were normalized using center log ratio transformation.

To further discriminate the factors influencing the diversity of the elephant microbiota at zoos multifactorial analysis on both α (Shanon extropy) and β (Bray Curtis) diversity metrics were used. The results revealed a significant influence of institution (*p* < 0.001), diet (*p* < 0.001, in particular provision of branches (*p* < 0.001)), daily activity (*p* = 0.037) but not from sex (*p* = 0.271) and age (*p* = 0.745) (Table 2). Similarly, diet (*p* = 0.001), daily activity (*p* = 0.006), and institution (*p* = 0.048) were found as influential factors on the community composition as measured by Bray Curtis’s distance (β diversity, Table 2). A trend was also seen for sex (*p* = 0.065) and age (*p* = 0.089). At the genera level, a total of 144 taxa were detected. The differential abundance analysis (Aldex method, based on Kruskal–Wallis) revealed 12 taxa that were significantly different among groups based on the diet provided (Figure 2). Besides, in elephants that did not have branches a shift from Fibrobacteres to Spirochaetes was seen. Significant differences were seen for some bacterial taxa ratios depending on daily activity, sex, and provision of branches (Supplementary Figure 2). Specifically, the higher daily activity (> 5 km), the higher abundance of Lentisphaerae (*p* = 0.004, Kruskall–Wallis) and Victivallaceae (*p* = 0.013), but the lower percentages of Synergistetes (*p* = 0.05), Verrucomicrobia (*p* = 0.04) and Ruminococcaceae (*p* = 0.01). Males exhibited significantly more bacteria in the phyla Spirochaetes (*p* = 0.07) and Synergistetes (*p* = 0.035). Verrucomicrobiaceae (*p* = 0.001), Acholeplasmataceae (*p* = 0.006), and Paenibacillaceae (*p* = 0.012) were overrepresented in elephants not being provided with browse on a daily basis (Supplementary Figure 2).

**Table 2:** Analysis of multiple factors contributing to the diversity of the microbiome of zoo elephants.

Alpha diversity (Shannon entropy): ANOVA				
Factor	sum_sq	*df*	*F*	Pr(>*F*)
Diet	23.1	5	68.15	0.000
Sex	0.1	1	1.40	0.271
Age	0.1	4	0.49	0.745
Provision of branches	11.1	1	164.53	0.000
Institution	45.8	6	112.74	0.000
Activity	0.9	3	4.62	0.037
Residual	0.5	8	NA	NA

Beta diversity (Bray Curtis): Adonis PERMANOVA					
Factor	sum_sq	df	F	R2	Pr(>F)
Diet	3.35	5	2.95	0.46	0.001
Sex	0.31	1	1.36	0.04	0.065
Age	1.07	4	1.18	0.15	0.089
Institution	0.32	1	1.39	0.04	0.048
Activity	0.37	1	1.62	0.05	0.006
Diet: sex	0.29	1	1.30	0.04	0.082
Diet: age	0.27	1	1.19	0.04	0.182
Residuals	1.36	6	NA	0.19	NA
Total	7.34	20	NA	1.00	NA

### 3.2. Microbiomes of African savanna elephants in zoos and the wild are different, but share a core

**Figure 3:**
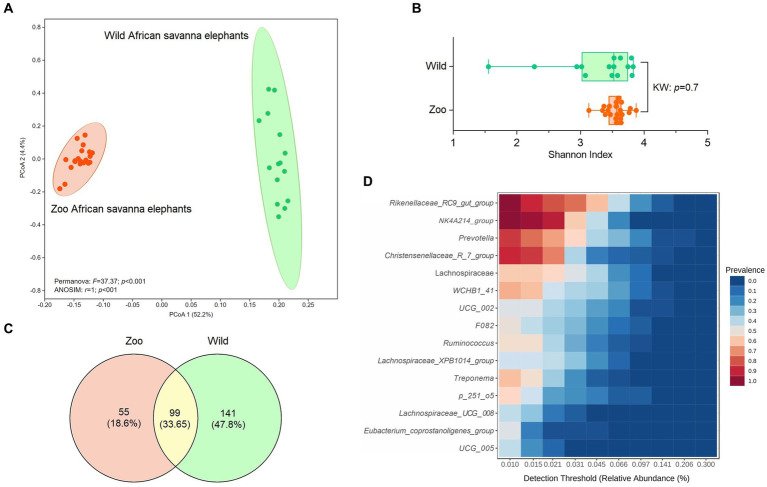
Comparison of microbiome composition at genera level of African savanna elephants living at zoos versus African savanna elephants living in the wild. **(A)** Principal coordinate analysis (PCoA) on Bray Curtis’s distance, compared with Permanova and ANOSIM tests. **(B)** comparison of Shannon entropy based on Kruskal–Wallis (KW) test (*p* > 0.05). **(C)** Venn diagrams indicate the proportion of unique and shared bacterial genera among wild and zoo elephants. **(D)** Detection of the core taxa among elephant microbiotas (present in both wild and captive elephants).

**Figure 4:**
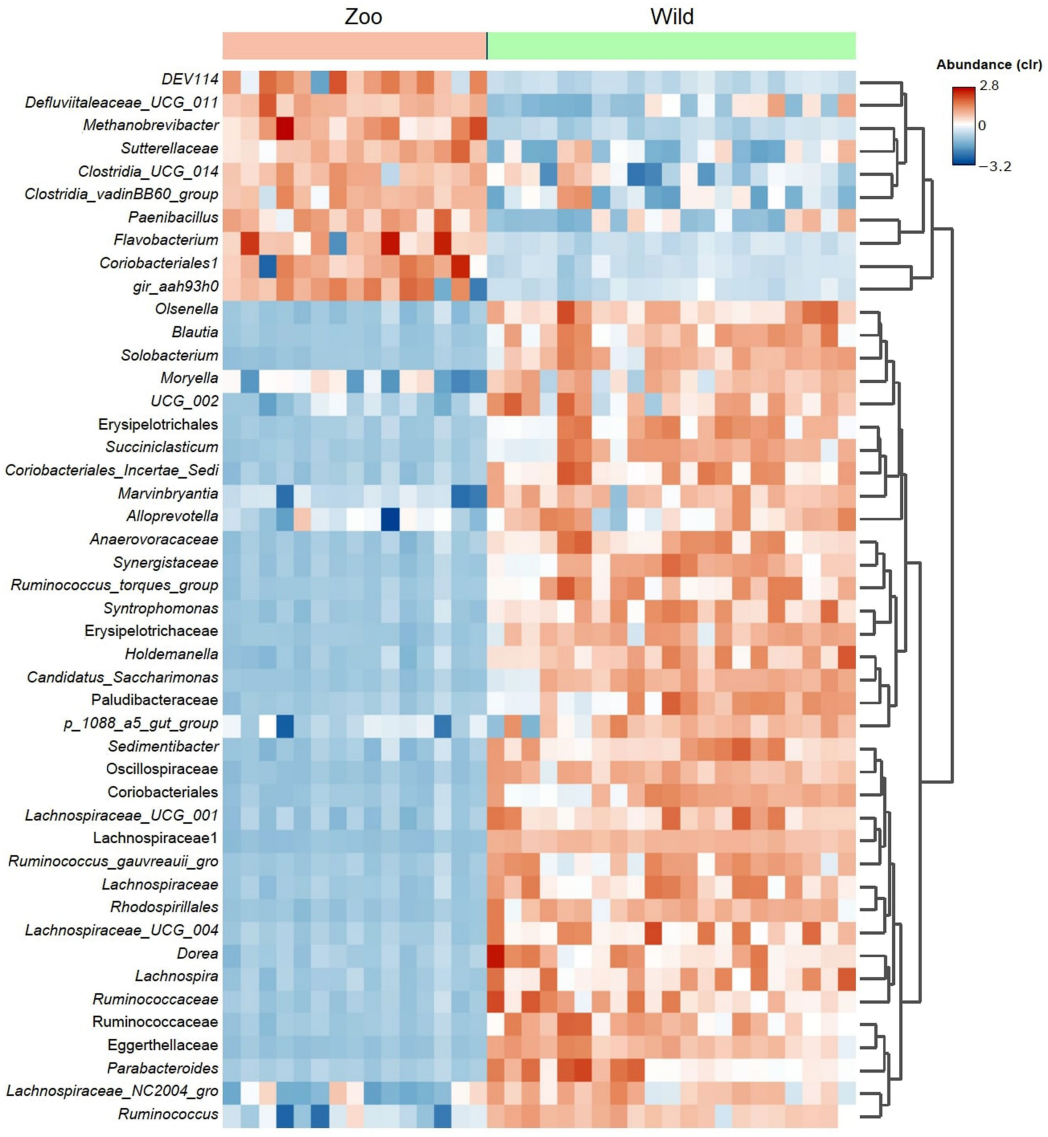
Heatmap shows differential abundant bacterial genera detected among microbiomes from African elephants living at zoos in the wild. The detection was performed using Aldex method (Welch test with Benjamini Hochberg correction). Only the top significant taxa (*p* < 0.001) are shown.

An average of 493,218.5 reads per sample (Budd et al., 2020) vs. 847,760.6 reads per sample for zoo elephants following sequencing of the 16S rRNA gene. The comparison of the microbiota composition (genera level) of African savanna elephants housed in French zoos and their similars of free living in Kenya revealed significant differences in taxonomic structure as confirmed by Permanova (*p* < 0.001) and Anosim (*p* < 0.01) tests on Bray Curtis distance (Figure 3A), although there were no differences in Shannon entropy (*p* = 0.7, Figure 3B). Among captive and free-ranging there were 99 taxa in common, which represented 33% of all the taxa detected (Figure 3C). A core set of taxa was shared for these animals wherever the living conditions (zoo or wilderness), including *Rikenellaceae_*RC9_gut group, *Prevotella*, and *Ruminococcus*, among others (Figure 3D). In addition, 46 bacterial genera with significantly different abundance (Aldex, Welch test with Benjamini Hochberg correction: *p* < 0.001) between zoo and wild elephants were detected (Figure 4). In particular, Ruminococcae and Lachnospiraceae were underrepresented in zoo elephants (Figure 4). The detailed relative abundance of the dominant bacterial families and genera in wild and zoo elephants is available in Supplementary Figures 3, 4, respectively.

**Figure 5:**
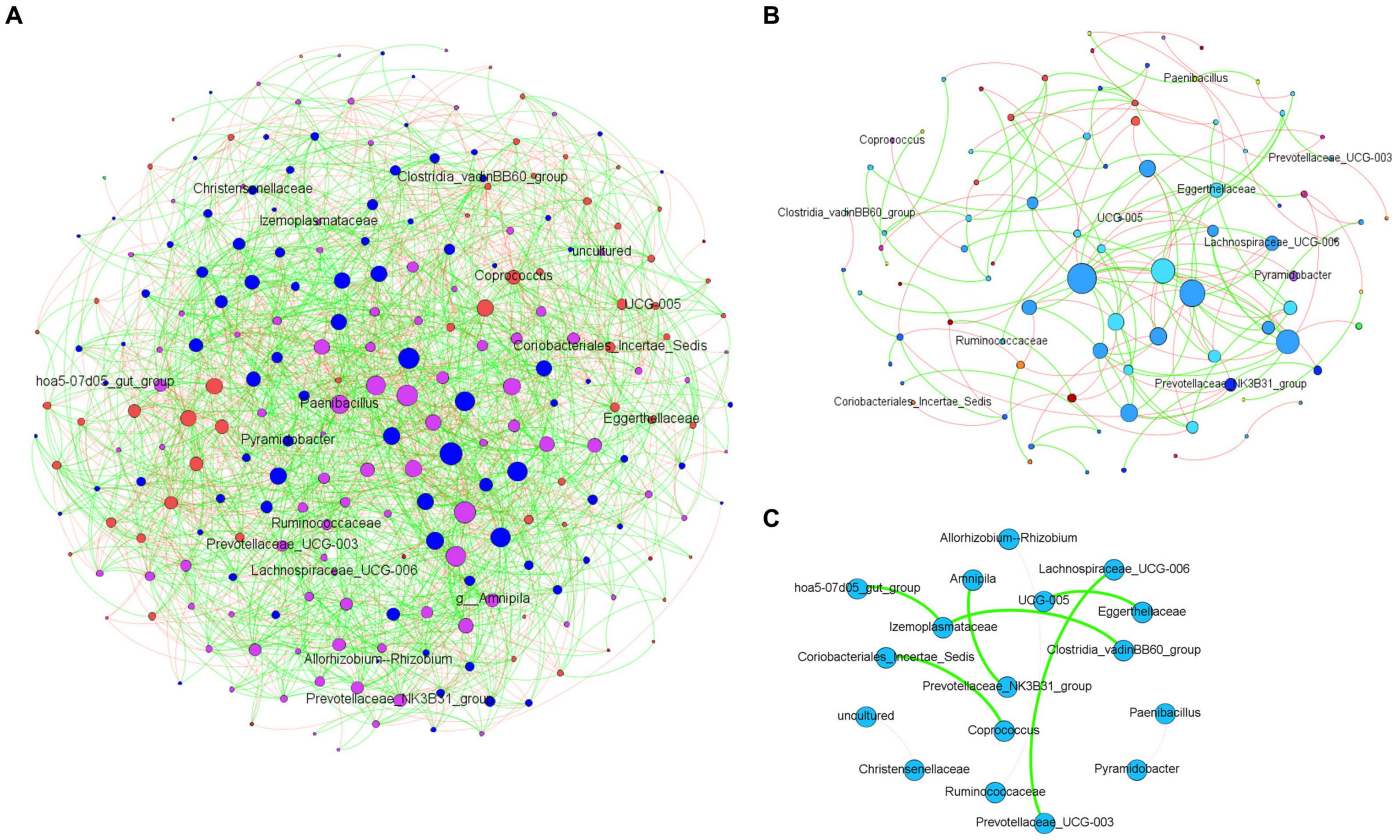
Microbial co-occurrence networks from African elephant microbiomes. Networks were inferred from **(A)** wild elephants and **(B)** zoo elephants. Co-occurrence networks were constructed using the SparCC method, including both significant negative (red edges) and positive (green edges) correlations (SparCC = 0.50). The node size indicated the eigen centrality of the nodes. **(C)** Core association network shows the common associations present across the microbiome of both wild and zoo elephants.

**Figure 6:**
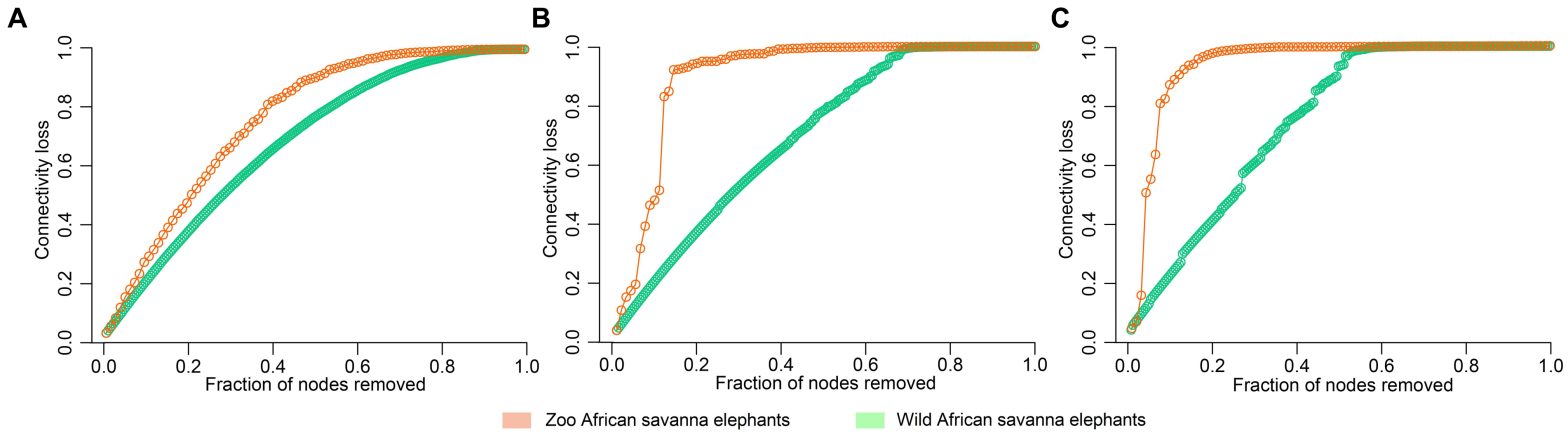
Robustness comparison of microbial network of elephants depending on their living condition. Loss of connectivity on the network depending on the fraction of nodes removed measured with **(A)** random, **(B)** degree, and **(C)** cascading method in wild (green) and zoo (orange) elephants.

The analysis of microbial co-occurrence networks detected clear differences between wild (Figure 5A) and captive elephants (Figure 5B), specifically because a higher number of nodes was detected in the free-ranging elephants, as well as in the connectedness of the nodes. Independently of the difference, a core association network (CAN) was detected within the two groups of elephants (captive vs. wild), represented by 9 connections (edges) among 17 taxa (nodes), from which three and six were negative or positive connections, respectively, (Figure 5C). The nodes represented in the CAN had a median to low relevance in the wild and zoo elephant microbial networks, respectively, and none of them constituted hub taxa in those networks. The smaller number of nodes needed to be removed to reach the maximum loss in connectivity indicated that the networks of zoo elephants exhibited lower robustness (ability of a network to maintain its functionality and structure in the face of perturbations) compared to those of wild elephants. This observation was consistent across various robustness methods, including random (Figure 6A), degree-based (Figure 6B), and cascading (Figure 6C) node removal methods.

## 4. Discussion

The study provides insights into the GM of managed African savanna elephants, offering a foundation for clinical comparisons, probiotic use, and transfaunation (Coverdale, 2016; Greene et al., 2019b), particularly for elephants facing changes due to captivity and anthropogenic activities (Moustafa et al., 2021). However, the scope of this study’s findings is limited to managed African savanna elephants, as GM composition varies across species, environments, and diets (Budd et al., 2020; Keady et al., 2021).

Limitations of this study encompass the small sample size (*n* = 21) and divergent sampling methods between French elephants (sterile collection of fresh dung boluses on FTA cards) and prior free-ranging data (20 g of fecal sample boiled, then stored at −20°C; Budd et al., 2020). Varied sample collectors necessitated a simple, rapid, and consistent method. FTA cards mitigated challenges of preserving fibrous samples and eliminated biases from freezing logistics (Supplementary Figure 1). While immediate freezing at −80°C is considered ideal, FTA cards showed high intraclass correlation coefficients for α- and β-diversities compared to freezing (Wang et al., 2018). Chosen for ease of use, repeatability, and moisture preservation (Stępień et al., 2019), FTA cards’ primary limitation was lower read counts compared to frozen dung boluses (Budd et al., 2020). An additional limitation lies in the different sequencing technologies used for the zoo and wild elephants, specifically Ion Torrent and Illumina, respectively. In our study, the Ion Torrent method targeted seven regions of the 16S gene. While Ion Torrent generally has lower sequencing depth and unique base-calling errors compared to Illumina, the multi-region targeting partially counterbalances these technological differences. To further ensure the rigor and validity of our comparative analysis, we implemented a consistent bioinformatics pipeline for both datasets, for instance (i) minimized noise through the DADA2 approach, (ii) standardized taxonomic assignments using the SILVA v138 database, and (iii) conducted network and statistical analyses based on centered log-ratio transformation (Aitchison, 1986), which made the data symmetrical.

Institutions (zoo of residence) significantly impacted β-diversity differences (Table 2), echoing US African savanna elephants’ findings (Keady et al., 2021). Different sampling methods were employed in that study (Keady et al., 2021), but husbandry practices, encompassing diet, training, and enrichment, uniquely shaped African savanna elephant GM (Table 2; Figures 1C, 2). Notably, regardless of pellet brand (horse, cattle, or elephant-designed), β-diversity remained similar, implying limited pellet impact (Figure 1C). Similar gut microbiomes among geographically proximate zoo elephants (Kartzinel et al., 2019; Keady et al., 2021) imply influential environmental and nutritional factors in GM, including hay types (Keady et al., 2021). Only one zoo in our study analyzed the analytical components of hay, so we did not include this parameter in the statistical analyses. However, variations in fruits, green vegetables, and seeds led to β-diversity differences, highlighting the significance of varied diets (Figure 1C).

Diet and daily activity are interrelated, with foraging and exploration increasing when branches are available (Lasky et al., 2021). In our study, diet, especially branch supply, significantly influenced zoo African savanna elephant GM. Elephants displaying higher daily activity exhibited elevated Lentisphaerae proportions, a pattern linked to better sleep in humans (Anderson et al., 2017). Augmented activity could offer improved sleep for managed elephants, potentially through increased access to branches in outdoor enclosures. This is all the more interesting since sleep behavior is recognized as a major indicator of elephant wellfare (Schiffmann et al., 2018). Factors like enclosure design complexity and NDF differences between diets with and without browse might also influence daily activity and GM (Clauss and Dierenfeld, 2008; Katole et al., 2014; Scott and LaDue, 2019; Wood et al., 2020). Encouragingly, mimicking wild diets with diverse branch types could enhance GM composition (Table 2), adjusting throughout the year according to tree species’ availability (Wood et al., 2020). Additionally, branches and browse can supply minerals and enhance vitamin uptake in elephants, but excluding pellets is not advised due to their role in meeting nutrient requirements, including vitamins D and E (Dierenfeld, 2006; Wood et al., 2020). Despite the benefits of branches, pellets play a vital role in balanced diets for captive elephants (Wood et al., 2020). Providing branches aligns with guidelines and enhances welfare by improving foraging and exercise opportunities (Bolechova et al., 2020; Lasky et al., 2021).

An influence of sex was seen on selected bacterial phyla, as Spirochaetes and Synergistetes were overrepresented in males. To the authors’ knowledge, it has not been assessed in adult African elephants so far, and hormonal differences between males and females may explain these differences. As Spirochaetes are overrepresented in horses fed exclusively on hay (Fernandes et al., 2021), male elephants may eat a higher proportion of hay than females. In other mammals, such as humans, the composition of intestinal microbiota differs over time between the sexes (Valeri and Endres, 2021), and in the closely related Asian elephants (*Elephas maximus*), the metabolizable energy intake is maintained from their diet across all seasons to reach sufficient needs to complete sex-specific physiological needs (Koirala et al., 2019). In addition, there is a link between hormones (including prolactin) and the structure of the gut microbiome in captive elephants (Keady et al., 2021). Such differences justify hindgut microbiome sex-specific research in African savanna elephants.

The GM of managed elephants was different than wild individuals, as significant differences were seen in its taxonomic structure between zoo and wild African savanna elephants (Figure 3A), with only 99 bacterial taxa in common (Figure 3C) and 46 bacterial genera with significantly different abundance (Figure 4). Digestive disorders may be more frequent in zoo elephants (Dumonceaux, 2006; Miller et al., 2015; Greene et al., 2019a; Scharling et al., 2021), as lower relative abundances of Ruminococcae and Lachnospiraceae have been consistently reported in horses with gastrointestinal disease (Costa et al., 2012; Schoster et al., 2017), and these bacterial families were underrepresented in zoo elephants (Figure 4). These members of the Firmicutes phylum are cellulolytic bacteria that degrade cellulose to produce energy-providing short-chain fatty acids (Kauter et al., 2019). Such bacteria are likely responsible for the production of specific enzymes responsible for colonic fermentation in elephants (Sthitmatee et al., 2011). Their lower abundance in zoo elephants may be linked to a lower diversity in diet items compared to the wild (Biru and Bekele, 2012; Pretorius et al., 2012; Mwambola et al., 2014; Sach et al., 2019), and with seasonal changes in food availability which is dramatically reduced in yearlong homogeneous pellet-based diet of captive-bred elephants (Codron et al., 2006; Dierenfeld, 2006; Clauss and Dierenfeld, 2008; Wood et al., 2020). This may explain markedly reduced bacterial occurrence networks in captive individuals as evidenced in Figure 5 and a markedly reduced network robusteness (Figure 6). Through zootechnical and nutritional modifications, increasing the robustness of microbiota networks in zoo elephants would bring them closer to those of wild elephants, resulting in a more resilient microbial community, better able to withstand disturbance, and improving their ability to adapt to changing conditions, respond to stressors and maintain optimal health.

Interestingly, our study revealed that despite differences in their environments, zoo elephants shared a core group of taxa with their wild counterparts. The shared “core microbiota” among zoo and wild African savanna elephants indicates a remarkable degree of consistency in certain microbial taxa across distinct environments. This finding suggests that while elephants in captivity settings and the wild experience contrasting diets and living conditions, there are underlying factors that drive the preservation of specific microbial communities. These core taxa, including Rikenellaceae, Prevotellaceae, Lachnospiraceae, and Ruminococcaceae, are not solely determined by host phylogeny, but also by the shared hindgut fermentation strategy common to herbivores like horses, rhinoceroses and smaller domestic species (Bian et al., 2013; Dougal et al., 2013; O'Donnell et al., 2017). This implies that the evolutionary heritage of hindgut fermenters contributes to the establishment of these core microbial communities, emphasizing the functional importance of these microbes in herbivorous digestion.

The discovery of core association networks (CANs) builds upon the concept of the “core microbiota” by revealing consistent patterns of microbial interactions. These CANs represent stable relationships that persist across different conditions (Röttjers et al., 2021), regardless of the varying dietary and environmental contexts experienced by zoo and wild elephants. The presence of these non-random associations in both captive and wild populations signifies meaningful microbial interactions that contribute to host health and functioning. The CANs provide insights into the coexistence and cooperation of microbial species within the gut ecosystem, contributing to the complex dynamics of hindgut fermentation and nutrient processing. The correlation between the shared core taxa and the CANs becomes particularly intriguing. The presence of certain microbial groups in the core microbiota may influence the formation of specific microbial associations within the CANs. For instance, the cellulolytic capabilities of members of the Ruminococcaceae (Froidurot and Julliand, 2022) family among the core taxa can shape interactions within CANs by affecting the breakdown of complex plant material. These interactions, in turn, might impact nutrient availability for the host (Froidurot and Julliand, 2022), and potentially contribute to the observed adaptations in digestive strategies.

## 5. Conclusion

This study holds significant implications for comprehending the impact of living environment on the gut microbiota (GM) of the endangered species *Loxodonta africana*, the African savanna elephant. The findings not only provide crucial insights for enhancing the management of these animals in zoos but also contribute to a relatively nascent area of knowledge: the intricate ecosystems constituted by host-associated microbiota.

It is evident from this research that, irrespective of their habitat, African savanna elephants share a fundamental core composition of gut microbiota, characterized by the prevalence of bacterial types commonly associated with hindgut fermenters, notably Rikenellaceae, Prevotellaceae, Lachnospiraceae, and Ruminococcaceae. However, a striking revelation emerges as zoo-dwelling elephants exhibit a discernible reduction in both bacterial diversity and intricate interactions within the structure of their hindgut microbiota, in comparison to their wild counterparts.

Zoo husbandry factors emerged as pivotal determinants shaping the composition of the microbiota in zoo elephants. Among these factors, diet and daily activity stand out prominently. The influence of these factors, particularly diet and its seasonal variations, can elucidate the observed differences between captive and wild individuals. To optimize the gut health of zoo elephants and to mirror their natural dietary patterns, it becomes imperative to provide a diverse array of branches that can be foraged throughout their enclosures. This dietary enrichment has the potential to positively impact the composition and diversity of the microbiota.

## Data availability statement

The datasets presented in this study can be found in online repositories. The names of the repository/repositories and accession number(s) can be found at: https://www.ncbi.nlm.nih.gov/, PRJNA939400 (SRX19520099, SRX19520098, SRX19520097, SRX19520096, SRX19520095, SRX19520094, SRX19520093, SRX19520092, SRX19520091, SRX19520090, SRX19520089, SRX19520088, SRX19520087, SRX19520086, SRX19520085, SRX19520084, SRX19520083, SRX19520082, SRX19520081, SRX19520080, SRX19520079) https://www.ncbi.nlm.nih.gov/, PRJNA587772.

## Ethics statement

Ethical approval was not required for the study involving animals in accordance with the local legislation and institutional requirements because the study required only the collection of fresh boluses of elephant dung. These samples were collected immediately after the animal’s defecation, in a totally non-invasive manner (on the ground). No animals were handled to collect these samples, and no animal’s daily routine was altered for the study.

## Author contributions

MT, BM, P-YM, AC-C, and AL conceived the study. P-YM acquired the data. DO, AM, and AW-C performed the bioinformatic and statiscal microbiome analyses, and visualized the results. LM-H curated the data. MT, DO, and AC-C drafted the first version of the manuscript. AW-C edited and corrected the manuscript. All authors revised and accepted the last version of the manuscript.
