# Supplementary material for: Conserved core microbiota in managed and free-ranging Loxodonta africana elephants
Source: Front Microbiol. 2023 Oct 4;14:1247719. doi: 10.3389/fmicb.2023.1247719 (PMC10582353; doi:10.3389/fmicb.2023.1247719)
Supplement: Supplementary file 1 [file Table_1.DOCX]

**Supplementary** **Table 1.** Shannon and Simpson indexes at all taxonomic levels, sorted by individual influence factors.

| Factors |  | Phylum | | Family | | Genus | | Species | |
| --- | --- | --- | --- | --- | --- | --- | --- | --- | --- |
|  |  | Shannon | Simpson | Shannon | Simpson | Shannon | Simpson | Shannon | Simpson |
| Zoo ID  *p* = 0.999 | 1 | 1.06 | 0.61 | 2.63 | 0.89 | 1.90 | 0.77 | 1.71 | 0.71 |
|  | 2 | 1.22 | 0.61 | 2.81 | 0.90 | 1.96 | 0.80 | 2.28 | 0.86 |
|  | 3 | 1.13 | 0.59 | 2.55 | 0.87 | 1.44 | 0.68 | 1.67 | 0.73 |
|  | 4 | **1.42** | **0.65** | **3.18** | **0.94** | **2.44** | **0.88** | **2.39** | **0.89** |
|  | 5 | 1.14 | 0.60 | 2.65 | 0.89 | 2.12 | 0.81 | 1.93 | 0.81 |
|  | 6 | 1.14 | 0.59 | 2.67 | 0.88 | 2.06 | 0.80 | 1.47 | 0.68 |
|  | 7 | 1.09 | 0.59 | 2.65 | 0.89 | 1.79 | 0.77 | 1.74 | 0.79 |
| Provision of browse  *p* = 1.00 | Br + | 1.13 | 0.60 | 2.67 | 0.89 | **1.97** | 0.78 | 1.67 | 0.72 |
|  | Br - | 1.13 | 0.60 | 2.71 | 0.89 | 1.85 | 0.78 | **1.90** | 0.81 |
| Daily activity  *p* = 0.850 | < 5km | 1.15 | 0.60 | 2.73 | 0.89 | 1.85 | 0.77 | **1.88** | 0.80 |
|  | > 5km | 1.16 | 0.60 | 2.66 | 0.88 | **2.03** | 0.80 | 1 .63 | 0.72 |
| Born  *p* = 1.00 | Captive | 1.14 | 0.61 | 2.67 | 0.89 | 1.93 | 0.78 | **1.86** | 0.77 |
|  | Wild | 1.13 | 0.59 | 2.70 | 0.89 | 1.94 | 0.78 | 1.60 | 0.72 |
| Sex  *p* = 0.705 | F | 1.10 | 0.59 | 2.66 | 0.89 | 1.91 | 0.78 | 1.57 | 0.71 |
|  | M | **1.19** | 0.61 | **2.71** | 0.89 | **1.98** | 0.78 | **2.01** | 0.79 |
| Age  *p* = 0.850 | < 20 yr | **1.16** | 0.61 | 2.69 | 0.89 | **2.00** | 0.79 | **1.90** | 0.77 |
|  | > 20 yr | 1.11 | 0.59 | 2.68 | 0.89 | 1.89 | 0.78 | 1.63 | 0.73 |
