# Supplementary figures and images for: Conserved core microbiota in managed and free-ranging Loxodonta africana elephants
Source: Front Microbiol. 2023 Oct 4;14:1247719. doi: 10.3389/fmicb.2023.1247719 (PMC10582353; doi:10.3389/fmicb.2023.1247719)

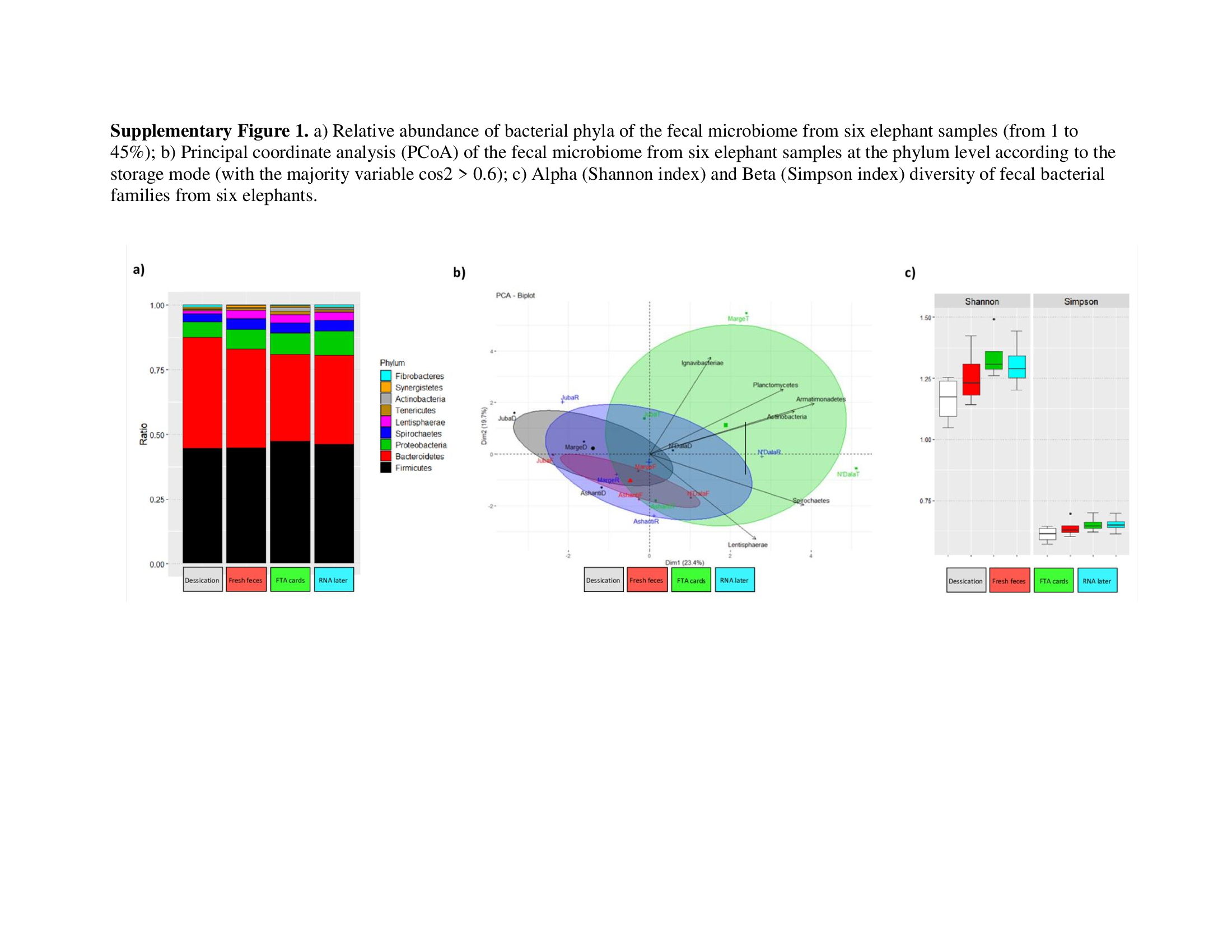

Supplement: Supplementary file 2 [file Image_1.jpg]

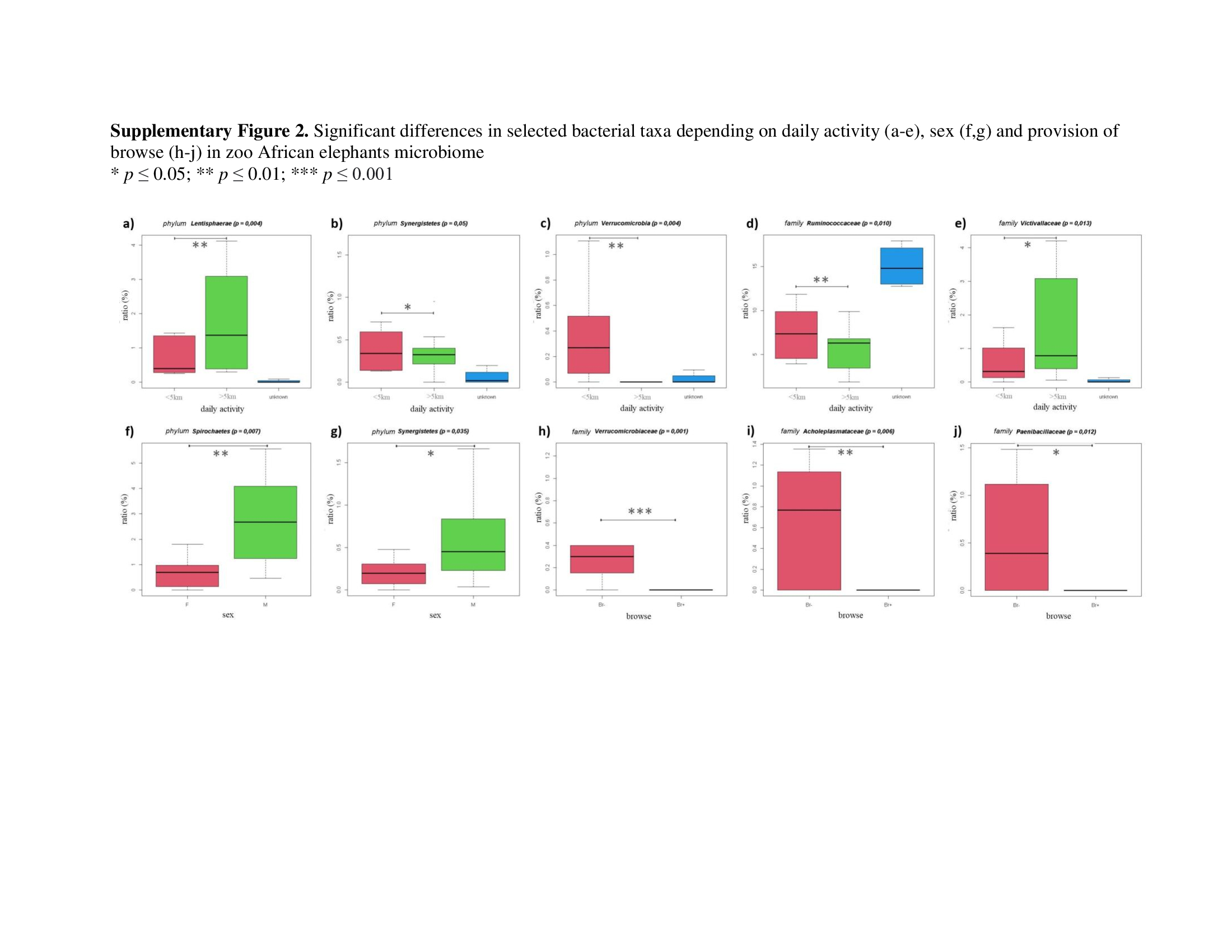

Supplement: Supplementary file 3 [file Image_2.jpg]

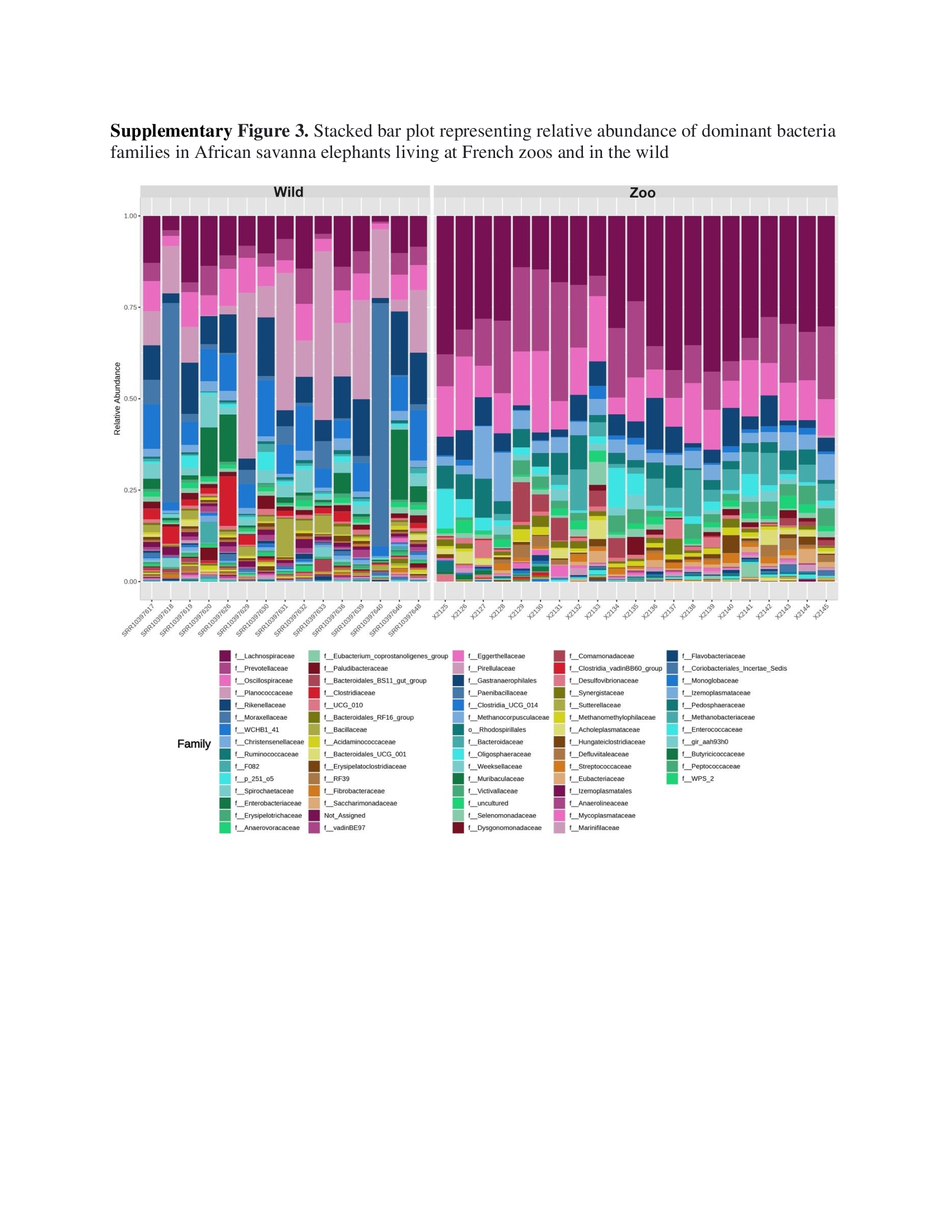

Supplement: Supplementary file 4 [file Image_3.jpg]

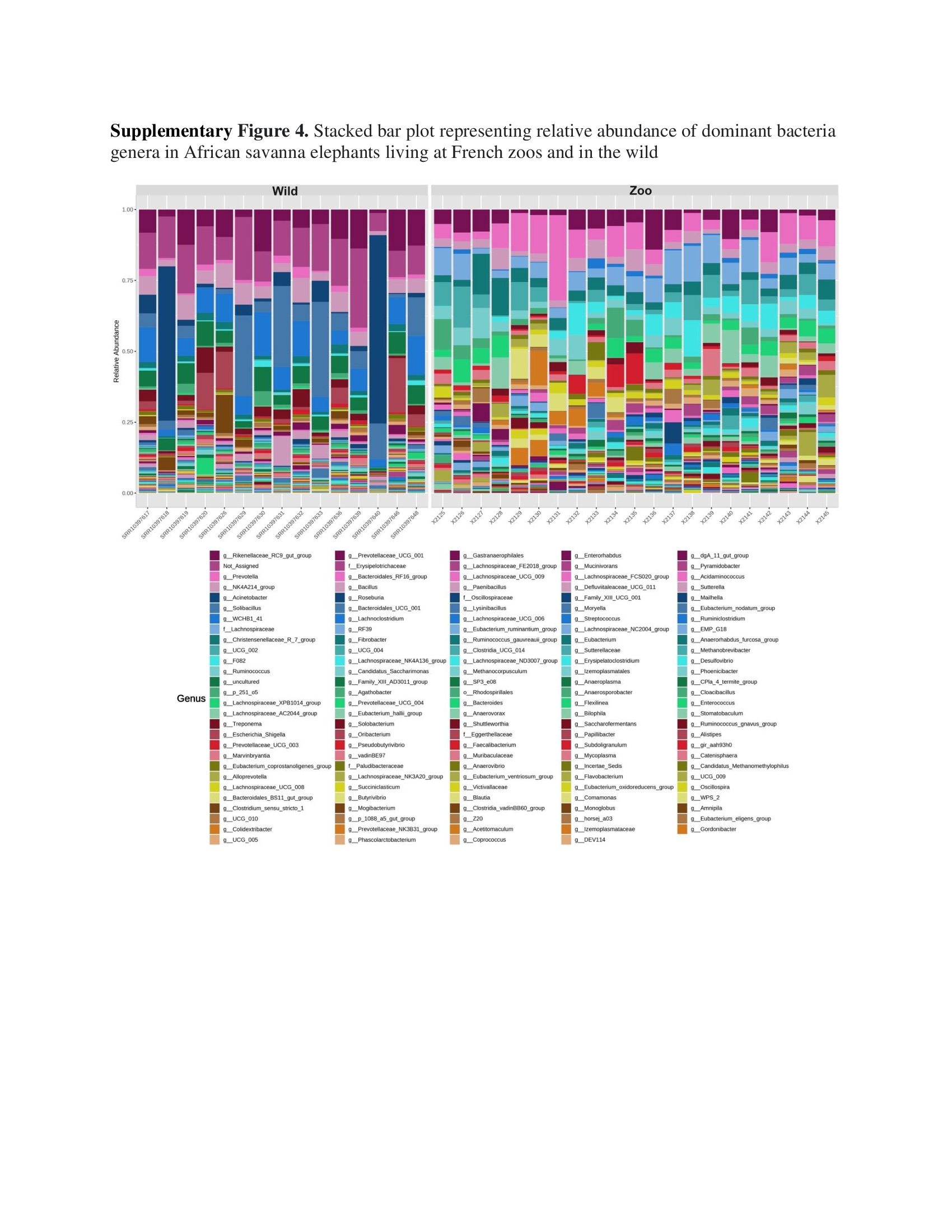

Supplement: Supplementary file 5 [file Image_4.jpg]
